# The Impact of Preexisting Cardiac Implantable Electronic Devices on the Safety of Transcatheter Tricuspid Valve Interventions: A Systematic Review and Meta-Analysis

**DOI:** 10.3390/jcdd13100478

**Published:** 2026-09-23

**Authors:** Yaman Almasri, Abdulrahman A. Sayed, Lama Alzahrani, Saad Mohammed Abdullah Alqahtani, Renad Saeed Algarni, Amro Badra, Majid Naif Alharbi, Ayman Al Sibai, Muath Alqahtani, Mohammed Alshareef, Adel Turki D. Alenezi, Saja Faisal Alzahrani, Bader Mansour Alotaibi, Ali Bakr, Muhammad Azam Shah

**Affiliations:** 1 College of Medicine, Alfaisal University, Riyadh 11533, Saudi Arabia; yalmasri@alfaisal.edu (Y.A.); aymanalsibairesearch@gmail.com (A.A.S.); 2 College of Pharmacy, Jazan University, Jazan 45142, Saudi Arabia; dr.abdulrahman987@gmail.com; 3 College of Medicine, AlMaarefa University, Riyadh 11597, Saudi Arabia; lamaalzahrani@yahoo.com; 4 Adult Cardiology Department, Armed Forces Hospital Southern Region, Khamis Mushait 62413, Saudi Arabia; saad5051995@gmail.com; 5 College of Medicine, King Saud bin Abdulaziz University for Health Sciences, Jeddah 22384, Saudi Arabia; ranoda.2.2002@gmail.com; 6 Prince Sultan Cardiac Centre—Qassim, Qassim Health Cluster, Buraydah 52366, Saudi Arabia; majedalharbi599@yahoo.com; 7 College of Medicine, King Khalid University, Abha 61421, Saudi Arabia; muath.a.alqahtani@gmail.com; 8 College of Medicine, Fakeeh College for Medical Sciences, Jeddah 23323, Saudi Arabia; alshmohammed2002@gmail.com; 9 Internal Medicine, Security Forces Hospital Riyadh, Northern Border University, Riyadh 11481, Saudi Arabia; adel.2019@yahoo.com; 10 College of Medicine, Umm Al-Qura University, Makkah 21955, Saudi Arabia; ssaj911@gmail.com; 11 College of Medicine, King Saud bin Abdulaziz University for Health Sciences, Riyadh 11481, Saudi Arabia; badr90490@outlook.com; 12 Faculty of Medicine, Al-Azhar University, Cairo 11884, Egypt; dralibakr93@gmail.com; 13 Department of Cardiology, King Fahad Medical City, Riyadh 11525, Saudi Arabia; azamshah165@hotmail.com

**Keywords:** tricuspid regurgitation, transcatheter tricuspid valve interventions, cardiac implantable electronic devices, systematic review, meta-analysis

## Abstract

Background: Tricuspid regurgitation (TR) increases mortality but remains undertreated due to high surgical risks. Transcatheter tricuspid valve interventions (TTVI) offer safer alternatives, improving functional status and quality of life. However, many candidates for TTVI have trans-tricuspid CIED leads that may interfere with procedural imaging and device deployment and may be exposed to dislodgement, entrapment, or jailing. Evidence regarding both clinical outcomes and postprocedural CIED function in this population remains limited. Existing trial evidence for this population is scarce, requiring further safety analysis. To our knowledge, this is the first meta-analysis to address the comparative risk of complications and residual TR in patients with vs. without CIEDs. Methods: We searched for relevant articles up to March 2026 from PubMed, Scopus, Cochrane Library, and Web of Science. Two independent reviewers extracted data from the selected studies, including baseline information, outcomes, comorbidities, hospitalization metrics, TR severity, and complications. All data analyses were performed using R version 4.4.1. Results: Ten studies comprising 5415 patients were included. Nine comparative studies contributed to the quantitative synthesis, whereas one single-arm study contributed only to the narrative synthesis of CIED-specific mechanical and electrical outcomes. In patients undergoing TTVI, across two studies, patients with CIEDs had a nominally lower risk of single-leaflet device attachment (RR 0.34, 95% CI 0.12–0.94; nominal *p* = 0.037); however, this exploratory finding should be considered hypothesis-generating. However, no statistically significant differences were observed between groups for cardiovascular death (RR 1.30, *p* = 0.815), device embolization (RR 1.62, *p* = 0.666), device malposition (RR 1.10, *p* = 0.909), stroke (RR 0.52, *p* = 0.335), hospitalization for heart failure (RR 1.02, *p* = 0.931), in-hospital mortality (RR 1.02, *p* = 0.959), major adverse events (RR 0.74, *p* = 0.635), major bleeding (RR 0.70, *p* = 0.187), or residual tricuspid regurgitation of mild (RR 0.98, *p* = 0.686) or moderate severity (RR 1.01, *p* = 0.646). CIED-specific outcomes were reported heterogeneously. Lead function was generally preserved after T-TEER in carefully selected patients, whereas studies of TTVR with jailed leads reported lead failure or malfunction ranging from 5.8% to 21% in individual cohorts. Conclusions: Among the comparative outcomes available for quantitative pooling, no statistically significant differences were detected between patients with and without CIEDs for most procedural and postprocedural outcomes. However, CIED-specific risks differed according to the intervention. Lead function was generally preserved after T-TEER in carefully selected patients, whereas lead jailing during TTVR was associated with clinically relevant lead malfunction in some cohorts. Therefore, the absence of significant differences in pooled clinical outcomes should not be interpreted as evidence that TTVI does not affect CIED function.

## 1. Introduction

Tricuspid valve disease, especially tricuspid regurgitation (TR), is a common disorder that has traditionally been considered a neglected condition and is frequently inadequately managed in clinical practice [1]. Untreated TR increases morbidity and mortality independently of pulmonary hypertension or pre-existing ventricular dysfunction [2]. Patients with severe TR present with right-sided congestive heart failure, manifesting as ascites, peripheral edema, diminished cardiac output, profound fatigue, reduced exercise capacity, and a poor long-term prognosis [2,3]. Although open heart surgery is considered definitive management, isolated tricuspid valve repair carries a higher perioperative mortality of 8% to 14% compared with other single valve procedures [1,4]. Given the notorious operative risks in this patient population, transcatheter tricuspid valve interventions (TTVI) have been developed to offer a new therapeutic approach for those refractory to medical therapy [2].

In the field of TTVI, several modalities are available, including transcatheter edge-to-edge repair (TEER), orthotopic transcatheter tricuspid valve replacement (TTVR), direct percutaneous annuloplasty, and heterotopic caval valve implantation (CAVI) [5,6]. TTVI has demonstrated a significant reduction in TR severity in the majority of treated patients [7]. Consequently, greater improvements have been observed in New York Heart Association (NYHA) functional class, increased Kansas City Cardiomyopathy Questionnaire (KCCQ) scores for quality of life and extended 6-min walk distances (6MWD) [3,7,8]. Moreover, successful transcatheter TR repair promotes positive right ventricular reverse remodeling, as indicated by reductions in right ventricular end-diastolic and end-systolic volumes and a decrease in systemic venous congestion [9]. Also, TTVI is considered to have a favorable periprocedural outcome, with lower 30-day mortality rates for TEER than TTVR [2]. However, bleeding complications vary by device, with TTVR demonstrating higher rates than TEER [2]. Additionally, TTVR may disturb the conduction system, making permanent pacemaker implantation necessary in up to 15% of patients [10].

Cardiac implantable electronic devices (CIEDs) comprise different therapeutic systems, including permanent pacemakers, implantable cardioverter defibrillators (ICDs), and cardiac resynchronization therapy (CRT) devices [11]. A substantial proportion of patients with severe TR have coexisting CIED leads that cross the tricuspid valve [12]. These transvalvular leads can cause or exacerbate valvular dysfunction through multiple mechanisms, such as direct mechanical leaflet impingement, leaflet perforation, chordal entanglement, fibrotic adhesion, and pacing-induced right ventricular remodeling [13]. Performing TTVI in patients with existing CIEDs can obscure accurate echocardiographic imaging and proper device positioning during the procedure [14]. Furthermore, TTVI may cause lead entrapment, which carries a high risk of lead entanglement or dislodgement, as well as potential interference with proper valve leaflet function or sealing [12].

Despite the potential advantages of TTVI, existing clinical trials have insufficiently included patients with cardiac implantable electronic devices CIEDs [15,16]. Therefore, direct comparisons assessing TTVI outcomes between CIED and non-CIED patients are largely limited to observational studies and small, single-center cohorts, which call for larger prospective validations [5,6,17]. Accordingly, this systematic review and meta-analysis aimed to compare procedural, clinical, and residual-TR outcomes following TTVI in patients with versus without preexisting CIEDs and to interpret these findings in the context of recognized CIED-specific mechanical and electrical complications.

## 2. Materials and Methods

This review followed the guidelines provided by the Preferred Reporting Items for Systematic Reviews and Meta-Analyses (PRISMA) and the Cochrane Handbook for Systematic Reviews of Interventions [18,19]. We registered the study protocol prospectively on the International Prospective Register of Systematic Reviews (PROSPERO) under the registration number: CRD420261428425.

### 2.1. Eligibility Criteria

Inclusion Criteria:

- Comparative studies reporting procedural, clinical, or residual-TR outcomes in CIED-positive and CIED-negative patients were eligible for quantitative synthesis. Single-arm TTVI cohorts were eligible only when they reported extractable CIED-specific mechanical or electrical outcomes and were included exclusively in the narrative synthesis.
- Studies reporting safety and efficacy data.

Exclusion Criteria:

- Non-English studies.
- Single-arm studies that did not report extractable CIED-specific mechanical or electrical outcomes.
- Studies lacking extractable outcome data.

### 2.2. Information Sources and Search Strategy

Preliminary scoping searches were conducted to refine the search terminology. The formal systematic search was subsequently performed in PubMed, Scopus, and Web of Science through March 2026. Then, a more focused search strategy integrating MeSH terms and associated keywords was systematically applied to PubMed, Scopus, and Web of Science up to March 2026. The specific search terms used for our review were: ((Tricuspid OR “Right atri* valve” OR “Right AV” OR TV) AND (“T-TEER” OR “edge-to-edge repair” OR Transcatheter OR Transjugular OR Transvalvular OR “TTVA” OR annuloplasty OR “TTVI” OR “TTVR” OR Intervention OR Replacement) AND (“CIED” OR “Cardiac Implantable Electronic Device” OR “TLE” OR “transvenous lead extraction” OR “CRTD” OR “resynchronization therapy” OR CRT OR Defibrillat OR “ICD” OR Pacemaker) AND (regurg* OR TR OR LTR OR Incompetence OR Insufficiency)).

### 2.3. Selection Process

To minimize bias, two reviewers independently evaluated the titles and abstracts of all records, masked to author information using predefined criteria. A third reviewer resolved any conflicts. The same two reviewers then assessed the full texts of eligible articles, comparing their decisions to resolve discrepancies.

### 2.4. Data Collection

Two independent reviewers extracted data from full-text articles into a shared Google spreadsheet using a predetermined set of variables in a two-step process. First, for each included trial, the study identifier and corresponding NCT number were recorded. The following data were extracted: number of patients and proportion of female patients, patient age, mean follow-up duration, KCCQ score for health-related quality of life; number and percentage of patients with NYHA functional class III or IV for functional status; functional capacity assessed by 6MWD in meters, operative risk measured by the EuroSCORE II and the TRI-SCORE, TR severity categorized as severe, massive, or torrential, with the number and percentage of patients in each category; and associated comorbidities, including atrial fibrillation (AF), hypertension (HTN), heart failure hospitalization, diabetes, and coronary artery disease (CAD), each reported as number and percentage. Second, for outcome measures, the following outcomes were extracted for analysis: incidence of cardiovascular death (CVD), device embolization, device malposition, heart failure hospitalization, in-hospital mortality, major adverse events (MAE), major bleeding, mild residual TR, moderate residual TR, overall mortality, single-leaflet device attachment (SLDA), and stroke.

CIED-specific outcomes were also extracted when reported, including lead dislodgement, lead entrapment or jailing, changes in pacing threshold, sensing amplitude or lead impedance, loss of capture, lead malfunction or failure, device reprogramming, and the need for lead revision, extraction, replacement, or an alternative pacing strategy. The timing of device interrogation and duration of device-specific follow-up were also recorded.

### 2.5. Outcome Measures

**The Risk of Complications of Transcatheter Tricuspid Valve Interventions (TTVI) in Patients with Versus Without Cardiac Implantable Electronic Devices (CIEDs):**

For complications, we compiled data related to single-leaflet device attachment risk, cardiovascular death, device embolization, device malposition, and stroke.

**Hospitalization Metrics After TTVI in Patients with Versus Without CIEDs:**

Hospitalization metrics included risk of hospitalization for heart failure, in-hospital mortality, major adverse events, and major bleeding risk.

**The Severity of Tricuspid Regurgitation (TR) After TTVI in Patients with Versus Without CIEDs:**

The risk of residual TR, both mild and moderate, was pooled from the included studies and compared between people with and without CIEDs.

**CIED-Specific Mechanical and Electrical Outcomes**

CIED-specific mechanical outcomes included lead dislodgement, entrapment or jailing, lead fracture, and the need for lead revision, extraction, or replacement. Electrical outcomes included changes in pacing threshold, sensing amplitude, impedance, loss of capture, lead noise, device reprogramming, and lead failure. Because definitions, denominators, follow-up durations, and device-interrogation protocols differed substantially among studies, these outcomes were synthesized narratively rather than pooled quantitatively.

### 2.6. Risk of Bias

Two reviewers independently assessed study quality, with disagreements resolved by a third reviewer. Because CIED status was not randomized in any included comparison, all CIED-positive versus CIED-negative analyses, including the subgroup analysis derived from the TRILUMINATE trial, were treated as observational cohort comparisons and assessed using the NIH Quality Assessment Tool for Observational Cohort and Cross-Sectional Studies. Single-arm studies included only in the narrative synthesis were assessed using the corresponding NIH tool [20].

### 2.7. Statistical Analysis

All analyses were conducted in R version R 4.4.1 using the meta package [21]. All outcomes were analyzed using the metabin function and prediction intervals. The data were presented as forest plots with pooled risk ratios (RR), 95% confidence intervals (CI), and weights. Pooled risk ratios were estimated using the Mantel–Haenszel method. For studies containing a zero cell, a continuity correction of 0.5 was applied only to the affected study. Studies with zero events in both groups were displayed in the forest plots for completeness but received zero weight and did not contribute to the pooled relative-risk estimate. A *p*-value of less than 0.05 was used to indicate statistical significance. Heterogeneity was assessed using the I^2^ statistic, with substantial heterogeneity defined as an I^2^ value greater than 40% and a χ^2^ *p*-value below 0.1. To address the heterogeneity stemming from varying operative factors, we applied a random effects model. The τ^2^ measure was estimated using the restricted maximum likelihood (REML) method. Given the clinical heterogeneity across TTVI modalities and study populations, random-effects models were retained for all outcomes. Prediction intervals based on fewer than four studies were considered statistically unstable and were not used for clinical inference. Because the outcome analyses were exploratory, no adjustment for multiple comparisons was applied; therefore, all *p*-values should be regarded as nominal.

## 3. Results

### 3.1. Study Selection

After searching three databases, we identified 2595 records. After removing 773 duplicates, 1822 unique records underwent title and abstract screening, of which 1787 were excluded. Thirty-five full-text reports were assessed for eligibility, and 25 were excluded: six reviews, two non-English reports, and 17 reports that did not meet the population or eligibility criteria. Ten studies were included in the systematic review. Nine comparative studies contributed to at least one quantitative meta-analysis, whereas Mekary et al. was a single-arm cohort included only in the narrative synthesis of CIED-specific outcomes. The flow diagram for study selection is shown in Figure 1.

### 3.2. Characteristics of the Included Studies

The baseline characteristics were derived from nine studies [5,8,16,17,22,23,24,25,26,27], conducted across the USA, Canada, Germany, Italy, Switzerland, France, Denmark, Georgia, and broader European and international multicenter sites, encompassing a total of 5415 patients. The cohort comprised 56.6% females (3066/5415), with an overall mean age of 78.78 ± 6.47 years. The weighted mean follow-up duration was 39.4 months (among 1455 patients with available data). Most patients were in NYHA functional class III or IV (81.1%; 4282/5279). The mean EuroSCORE II was 5.54 (4193 patients), and the mean TRI-SCORE was 6.22 (3723 patients). Among the 4241 patients with complete tricuspid regurgitation severity data, the distribution was severe in 38.1% (1614/4241), massive in 34.8% (1477/4241), and torrential in 22.7% (963/4241). Comorbidities were common: atrial fibrillation in 83.5% (4523/5415), hypertension in 75.0% (3557/4741), heart failure hospitalization in 35.1% (1783/5074), diabetes in 22.8% (1021/4476), and coronary artery disease in 40.3% (1514/3761). The mean 6-min walk distance was 244.3 ± 126.4 m (3889 patients). A complete depiction of baseline characteristics is displayed in Table 1.

### 3.3. Risk of Bias

All ten included studies were assessed using the NIH Quality Assessment Tool for Observational Cohort and Cross-Sectional Studies. Although the parent TRILUMINATE trial was randomized, the CIED-positive versus CIED-negative comparison reported by Naik et al. was treated as an observational subgroup analysis because CIED status was not randomized. Overall, seven studies were rated as fair quality, two as good quality, and one as poor quality (Table S1).

### 3.4. Results of Syntheses

The Risk of Complications of Transcatheter Tricuspid Valve Interventions (TTVI) in Patients with Versus Without Cardiac Implantable Electronic Devices (CIEDs):

Based on data provided by two studies, the CIED group had a significantly lower risk of single leaflet device attachment (SLDA; RR 0.34, 95% CI: 0.12–0.94, *p* = 0.037; Figure 2). In contrast, the risk of cardiovascular death (CVD) did not significantly differ between the group with CIED and those without (RR 1.30, 95% CI: 0.15–11.63, *p* = 0.815; Figure 3). Similarly, the risk of device embolization (RR 1.62, 95% CI: 0.18–14.48, *p* = 0.666; Figure 4), device malposition (RR 1.10, 95% CI: 0.21–5.70, *p* = 0.909; Figure 5), and stroke (RR 0.52, 95% CI: 0.14–1.97, *p* = 0.335; Figure 6) showed no statistically significant differences between the two groups.

Hospitalization Metrics After TTVI in Patients with Versus Without CIEDs:

Pooled data from three studies could not detect any statistically significant difference between those with CIED and those without in terms of the risk of hospitalization for heart failure (RR 1.02, 95% CI: 0.67–1.55, *p* = 0.931; Figure 7). Likewise, in-hospital mortality (RR 1.02, 95% CI: 0.46–2.29, *p* = 0.959; Figure 8), major adverse events (MAEs; RR 0.74, 95% CI: 0.22–2.54, *p* = 0.635; Figure 9), and major bleeding (RR 0.70, 95% CI: 0.42–1.19, *p* = 0.187; Figure 10) were statistically comparable between the two groups. This lack of statistical significance extended to all-cause mortality (RR 1.11, 95% CI 0.68–1.80; *p* = 0.679; Figure 11).

The Severity of Tricuspid Regurgitation (TR) After TTVI in Patients with Versus Without CIEDs:

Data pooled from four studies revealed that, after TTVI, the two groups experienced a statistically similar risk of mild (RR 0.98, 95% CI: 0.90–1.07, *p* = 0.686; Figure 12) and moderate residual TR (RR 1.01, 95% CI: 0.96–1.07, *p* = 0.646; Figure 13).

### 3.5. CIED-Specific Mechanical and Electrical Outcomes

CIED-specific outcomes differed according to the type of transcatheter intervention. Among selected patients undergoing T-TEER, Naik et al. reported no lead revisions, removals, or replacements during follow-up. Goebel et al. reported no lead malfunctions, although systematic preprocedural and postprocedural device interrogation was not mandated by the study protocol. In the EuroTR analysis by von Stein et al., paired assessments showed generally stable pacing thresholds, sensing, and impedance after T-TEER, and no lead revisions were required.

Following transcatheter tricuspid annuloplasty, Wrobel et al. reported one acute right ventricular lead dislodgement among 41 CIED carriers, requiring emergency implantation of a leadless pacemaker. Among the 24 patients with standardized preprocedural and postprocedural interrogation, right ventricular pacing thresholds and impedance remained stable, although statistically significant changes in some sensing and atrial impedance measurements were observed without clinically relevant device dysfunction.

CIED-related complications were more clinically relevant after orthotopic TTVR with lead jailing. Braunstein et al. reported acute lead dislodgement in one of 17 patients with jailed transvalvular leads, as well as a transient increase in pacing threshold in another patient. So et al. reported device-parameter deterioration requiring reprogramming in two of 22 patients with available device follow-up, without lead replacement or extraction. In the TRIPLACE registry, lead failure occurred in 6 of 104 patients with jailed leads (5.8%) over a median follow-up of 183 days. Mekary et al. reported lead malfunction in 3 of 14 patients with jailed leads (21%); two required lead revision, and one revision was complicated by infection followed by death. Because the definitions and follow-up periods differed considerably, these findings were not quantitatively pooled.

## 4. Discussion

A nominally lower risk of single-leaflet device attachment was observed among patients with CIEDs. However, this estimate was based on only two studies, had a confidence interval close to unity, and was not adjusted for multiple comparisons. It should therefore be regarded as hypothesis-generating rather than evidence that CIEDs protect against single-leaflet device attachment.

Although no statistically significant differences were detected between patients with and without CIEDs for most pooled procedural and postprocedural outcomes, these findings should not be interpreted as demonstrating equivalence between the groups or as evidence that TTVI does not affect CIED function. The quantitative analysis primarily evaluated clinical and procedural outcomes, including mortality, heart failure hospitalization, bleeding, stroke, device-related procedural complications, and residual TR. In contrast, CIED-specific mechanical and electrical outcomes were reported using heterogeneous definitions, denominators, device-interrogation protocols, and follow-up periods across the included studies [8,16,17,23,24,25,26,27]. Therefore, the clinical implications of a preexisting CIED should be interpreted according to the type of transcatheter intervention and the nature of the interaction between the transvalvular lead and the implanted tricuspid device.

In an echocardiography database analysis, Offen et al. reported that the presence of CIEDs was associated with a twofold increase in the risk of tricuspid regurgitation (TR) of moderate or worse severity (95% CI: 1.81–1.99). Moreover, they found an independent association between CIEDs and all-cause/cardiovascular death; however, mortality did not significantly differ between the CIED and non-CIED groups [28]. In response to this concern, Deharo et al. provided a statement from the European Society of Cardiology addressing the potential concerns regarding TTVI in patients with a preexisting CIED. Four approaches were evaluated in this document, including transcatheter edge-to-edge repair (TEER) and transcatheter tricuspid valve replacement (TTVR). The authors indicated that, with TEER, despite data showing the safety and efficacy of this approach in improving quality of life, further research is required to confirm the feasibility of this approach across diverse patient populations given the fact that this study was done to evaluate TEER in patients with specific anatomic criteria. In addition, more nuanced data remain necessary to determine the impact of a preexisting CIED that is causative of TR, and those of CIEDs that are not the cause of TR and are away from the grasping zone [6].

The presence of a CIED causes challenges during transcatheter interventions as the leads of the device physically and visually complicate the procedure. Echocardiographic shadowing artifacts caused by the leads make it difficult to clearly visualize the tricuspid leaflets [5,8,27]. Mechanically, the physical presence of the lead can impede the manipulation of the delivery system and devices within the right atrium, making it technically difficult to effectively grasp the leaflets or deploy clips [5,22,27]. In transcatheter edge-to-edge repair (TEER), operators may be forced to perform suboptimal placement of clips around the lead to navigate these constraints [24]. Furthermore, physical manipulation of the valve and delivery catheters carries a direct risk of causing acute lead dislodgement, which can result in immediate loss of pacing capture and require emergency pacemaker implantation [16,23,25].

The available evidence suggests that CIED function is generally preserved following T-TEER in carefully selected patients. In the TRILUMINATE analysis, Naik et al. reported no lead revisions, removals, or replacements during follow-up [24]. Similarly, Goebel et al. reported no CIED malfunction after T-TEER; however, device interrogation was not mandated by the study protocol, and subclinical or delayed electrical abnormalities could therefore not be excluded [8]. In the EuroTR analysis, von Stein et al. found that pacing thresholds and sensing remained stable following T-TEER, with only a modest change in impedance and no required lead revisions [27]. These findings should nevertheless be interpreted in the context of patient selection because patients with severe lead–leaflet interaction, inadequate imaging, or anatomy considered unsuitable for leaflet grasping may not have undergone T-TEER. Transcatheter annuloplasty also avoids permanent lead entrapment, but acute complications remain possible. Wrobel et al. reported complete right ventricular lead dislodgement in one of 41 CIED carriers, resulting in imminent third-degree atrioventricular block and requiring emergency leadless pacemaker implantation; among patients with paired device interrogations, right ventricular pacing thresholds and impedance otherwise remained stable [16].

During TTVR, an existing trans-tricuspid lead can become entrapped between the rigid frame of the new prosthetic valve and the native tricuspid annulus. This entrapment creates severe short- and long-term risks, such as life-threatening sepsis if device endocarditis occurs. This is often due to the virtual impossibility of fully extracting an entrapped device via a transvenous approach [17,29]. In a survival analysis, Traynor et al. found that there was no statistically significant difference between those who experienced a jailed CIED lead vs. those who did not (HR 1.36, 95% CI: 0.82–2.25) over a period of 12 months. In addition, they found that in patients who underwent TTVR, the jailed lead interfered with the device’s radial force distribution and impaired the seal between the prosthetic valve and the native annulus, resulting in significantly higher rates of moderate-to-severe paravalvular leaks. Furthermore, they analyzed the factors responsible for lead failure and found that prostheses that are large in size (HR 1.15, *p* = 0.048) and increased oversizing in systole (HR 1.12, *p* = 0.001) both increase the risk of lead failure [26]. The physical compression of the lead by the valve frame can cause chronic mechanical stress, insulation breaches, conductor fractures, and micro-dislodgment [23,25,26]. This can manifest months after the procedure as significant increases in pacing thresholds or loss of function, an especially detrimental outcome for pacemaker-dependent patients [17].

In contrast to T-TEER and annuloplasty, orthotopic TTVR may permanently jail a transvalvular lead between the prosthetic frame and the native annulus, creating distinct mechanical and electrical risks. Braunstein et al. reported acute right ventricular lead dislodgement requiring leadless pacemaker implantation in one patient, whereas the remaining preexisting devices demonstrated normal function during short-term follow-up [23]. In the study by So et al., two of 22 patients with available postprocedural interrogation exhibited worsening device parameters. In one patient, the pacing threshold began to increase four months after TTVR and required device reprogramming, although no lead revision, replacement, or extraction was required during follow-up [25]. In the multicenter TRIPLACE registry, lead failure occurred in six of 104 patients with jailed leads, corresponding to a rate of 5.8% over a median follow-up of 183 days. Lead failure was defined as structural or electrical malfunction requiring insertion of a new lead or CIED, and management included coronary sinus pacing, leadless pacemaker implantation, extraction of a jailed lead, or placement of a new right ventricular lead through the transcatheter valve [26]. Mekary et al. reported complications in three of 14 patients with jailed leads during a mean follow-up of 10.5 months, corresponding to a rate of 21%. Two patients required revision of fractured leads, and one revision was complicated by device infection, sepsis, and subsequent death [17]. Although these rates should not be directly compared because of differences in sample size, valve platforms, outcome definitions, and follow-up duration, they demonstrate that the absence of significant differences in mortality, hospitalization, or residual TR does not establish preservation of lead function following TTVR.

These findings highlight the importance of distinguishing successful valve implantation from preservation of long-term CIED function. Mechanical compression of a jailed lead may produce microdislodgement, insulation damage, conductor fracture, lead noise, altered impedance, increasing pacing thresholds, loss of capture, or delayed lead failure [17,23,25,26]. Furthermore, an entrapped lead may be extremely difficult or impossible to remove through a conventional transvenous approach if infection or lead failure subsequently develops. This concern is particularly important in pacemaker-dependent patients, in whom deterioration in lead function may have immediate clinical consequences. Consequently, decisions regarding lead extraction, lead preservation, or conversion to a valve-sparing pacing strategy should be individualized and made by a multidisciplinary heart team that includes structural interventionalists, electrophysiologists, cardiac imaging specialists, lead-extraction specialists, and cardiac surgeons [6,17,26,30].

Deharo et al. emphasized that all patients with a CIED lead crossing the tricuspid valve who are planned for a TTVI must be evaluated by a multidisciplinary cardiology team. This team should include an interventional cardiologist with TTVI expertise, a cardiac surgeon, a lead extraction specialist, and a cardiac imaging specialist [30]. When weighing the risks and benefits of transvenous lead extraction (TLE), aspects to be considered include patient’s life expectancy, comorbidities, and the risk of causing serious complications or damaging the tricuspid leaflets and subvalvular apparatus during extraction. TLE is generally favored for leads that have been implanted for less than 10 years or when advanced imaging confirms that TR is related to the CIED lead. In addition, they indicated that it is imperative to consider the type of CIED used, its system, frequency of therapy, and dependency status for the patient to avoid acute dislodgment or damage of the ventricular lead due to catheter manipulation during TTVI. For pacemaker-dependent patients, the device should be programmed to an asynchronous mode just before the procedure to prevent inappropriate pacing inhibition from sensing interferences. Electrical parameters must be reassessed immediately after the procedure to detect any acute lead dysfunction, and remote monitoring should be utilized long-term to catch any delayed malfunctions. They advocated for the installation of preventive or bailout temporary pacing strategies that do not cross the tricuspid valve for patients who are CIED-dependent, such as preemptive coronary sinus lead placement or emergency left or right ventricular wire pacing [31,32]. Standard RV temporary pacing leads should explicitly be avoided during TTVR because positioning and retrieving them around the new transcatheter valve can be challenging [6].

Complications associated with CIEDs should also be considered beyond their direct interaction with a transcatheter tricuspid device. Ceresa et al. described right ventricular perforation following pacemaker implantation, illustrating that transvenous lead implantation itself may rarely result in serious mechanical complications requiring multidisciplinary management [33]. Calvagna et al. reported embolization and retrieval of an anchoring sleeve during transvenous lead extraction, highlighting the technical complexity and potential complications associated with lead-removal procedures [34]. These reports do not directly assess comparative outcomes following T-TEER or TTVR and should therefore be considered contextual evidence rather than evidence that CIEDs independently worsen TTVI outcomes. Nevertheless, they emphasize the need to consider the lifetime management of the complete CIED system, including implantation-related complications, lead–valve interaction, possible lead jailing, device infection, long-term surveillance, and the feasibility and risks of subsequent extraction.

To our knowledge, this is the first meta-analysis to gauge the effects of CIEDs on various outcomes post-TTVI. Several limitations require consideration. Most included analyses were observational comparisons, even when the parent study was a randomized trial. The number of studies contributing to individual pooled outcomes was small, and several estimates had wide confidence intervals. CIED-specific outcomes were inconsistently defined, and device interrogation was not systematically required in all studies. Device-function data were available only for subsets of patients in some cohorts, creating a risk of ascertainment bias. Follow-up was frequently short and may not capture delayed fracture, insulation failure, rising pacing thresholds, infection, or progressive lead dysfunction. In addition, the clinical implications differ substantially between T-TEER, annuloplasty, and TTVR, limiting the interpretation of analyses that combine these modalities. Finally, potential overlap between large multicenter registries must be excluded before the same patients are entered into more than one pooled comparison.

## 5. Conclusions

Current evidence did not demonstrate statistically significant differences between patients with and without CIEDs for most pooled procedural and clinical outcomes following TTVI. Nevertheless, these findings do not establish equivalence or demonstrate that CIED function is unaffected. Device-related risks appear to be modality-specific: lead function was generally preserved after T-TEER in carefully selected patients, whereas permanent lead jailing during TTVR was associated with lead malfunction, failure, and the need for revision in some cohorts. Patients with transvalvular CIED leads therefore require multidisciplinary assessment, modality-specific procedural planning, peri-procedural device interrogation, and long-term surveillance. Prospective studies using standardized definitions and systematic reporting of electrical and mechanical lead outcomes are needed.

## Figures and Tables

**Figure 1:**
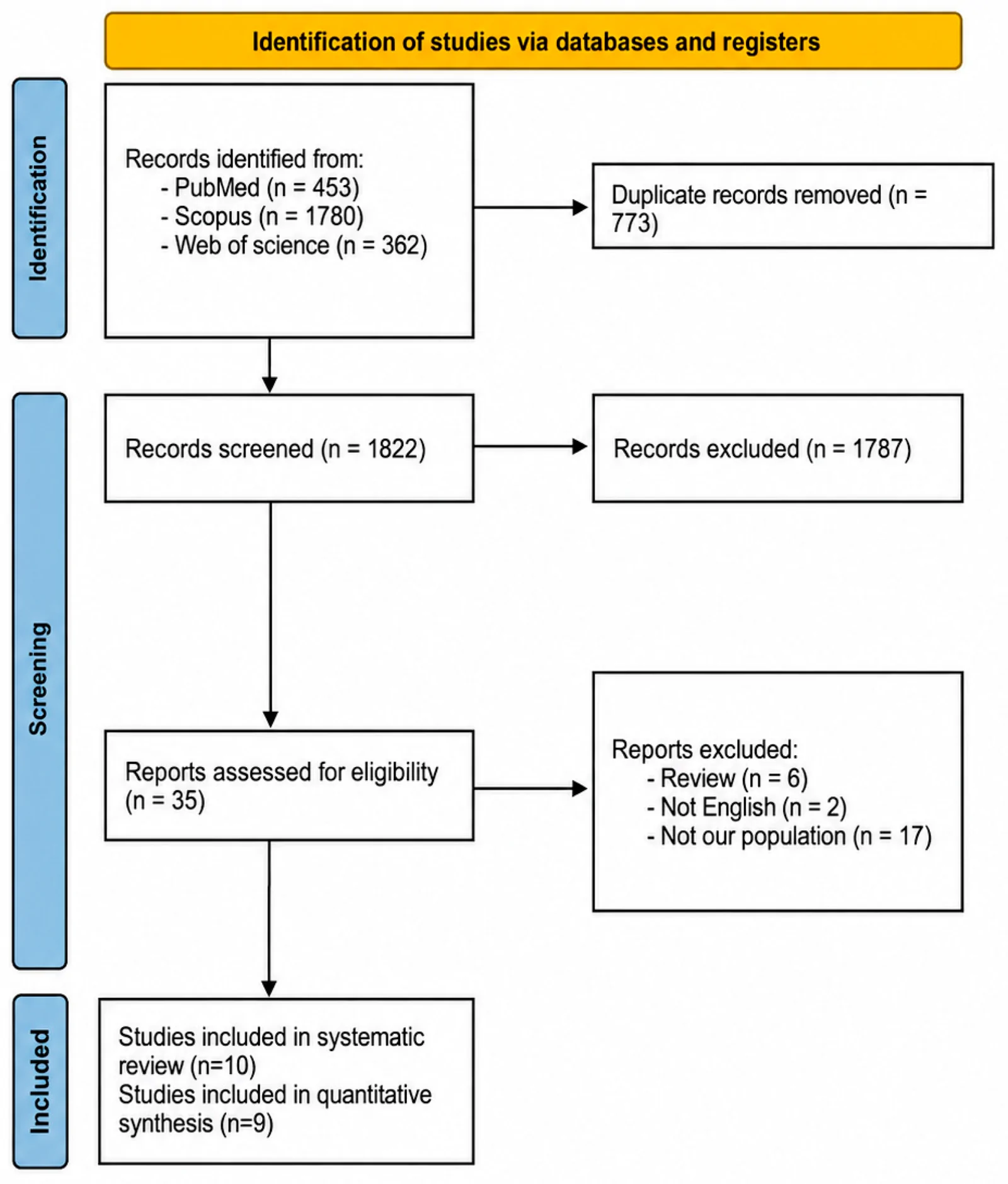
PRISMA flow diagram of study selection.

**Figure 2:**
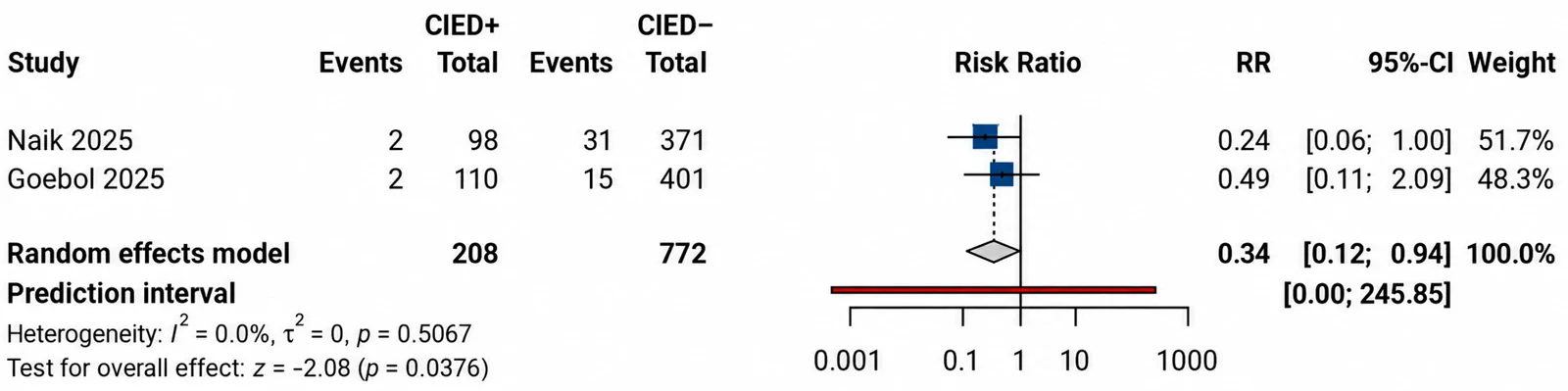
Forest plot of single-leaflet device attachment risk comparing CIED versus no CIED.

**Figure 3:**
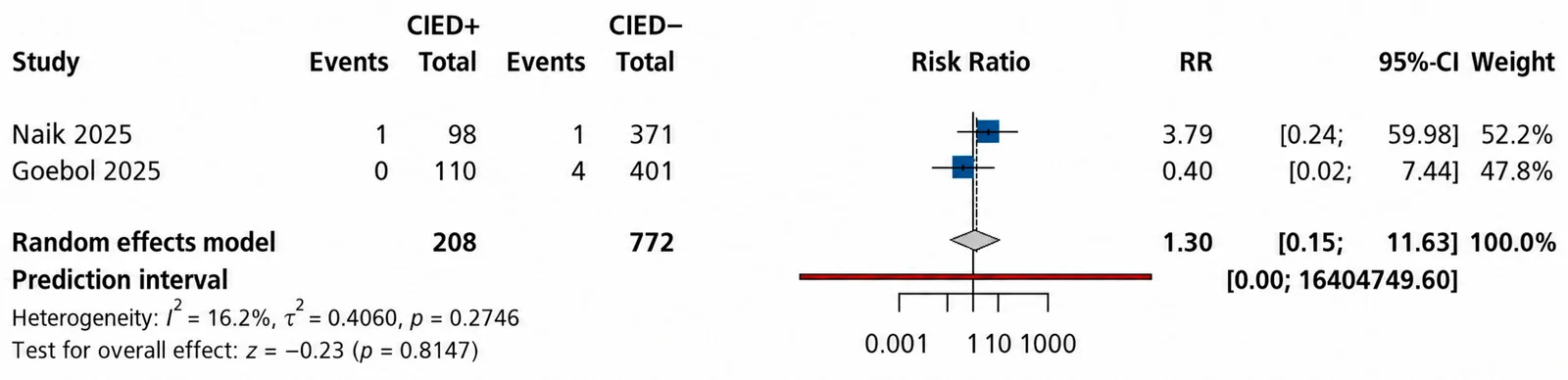
Forest plot of cardiovascular death risk comparing CIED versus no CIED.

**Figure 4:**
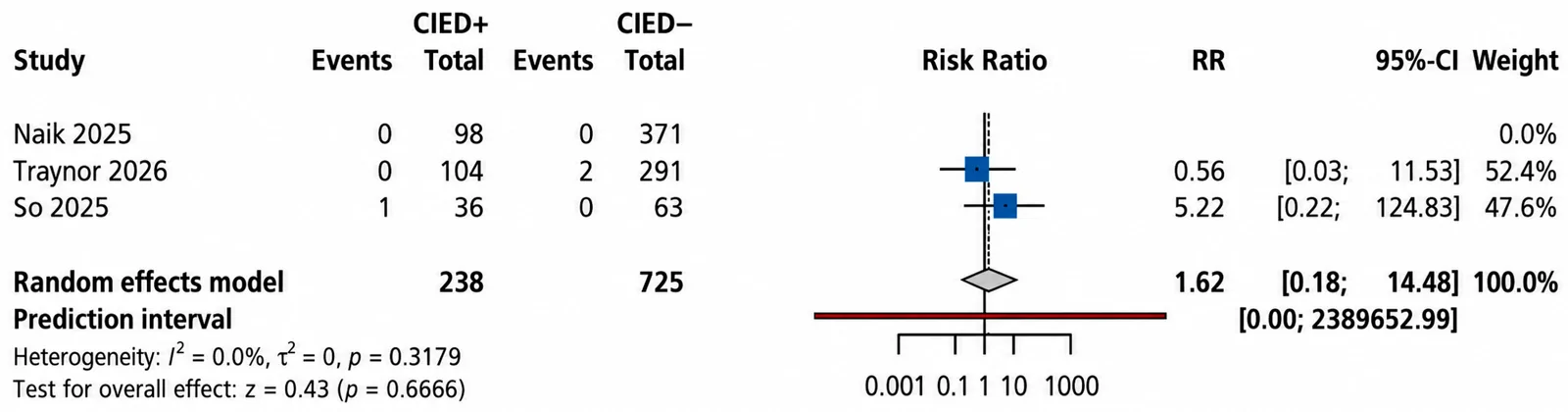
Forest plot of device embolization risk comparing CIED versus no CIED.

**Figure 5:**
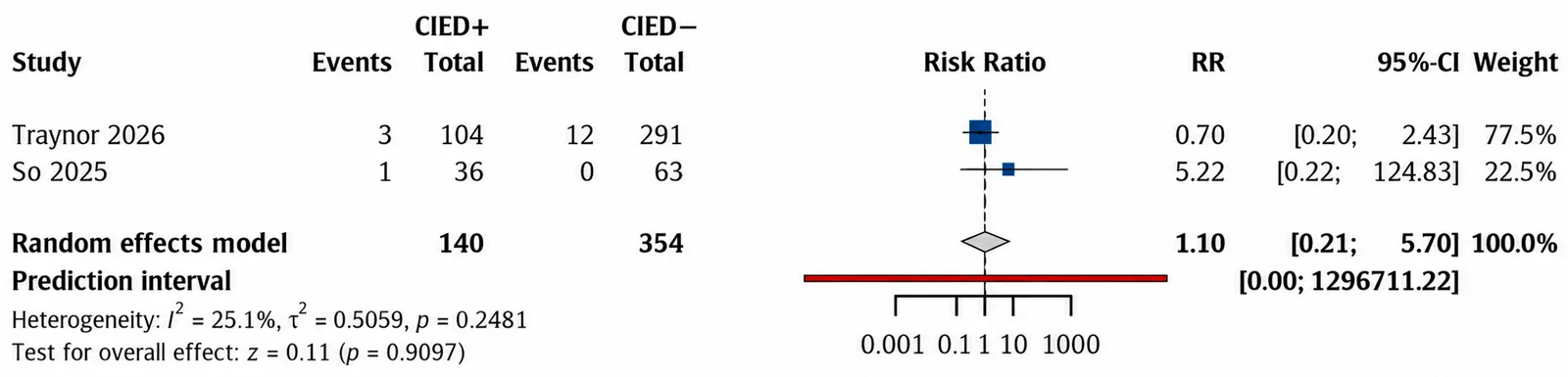
Forest plot of device malposition risk comparing CIED versus no CIED.

**Figure 6:**
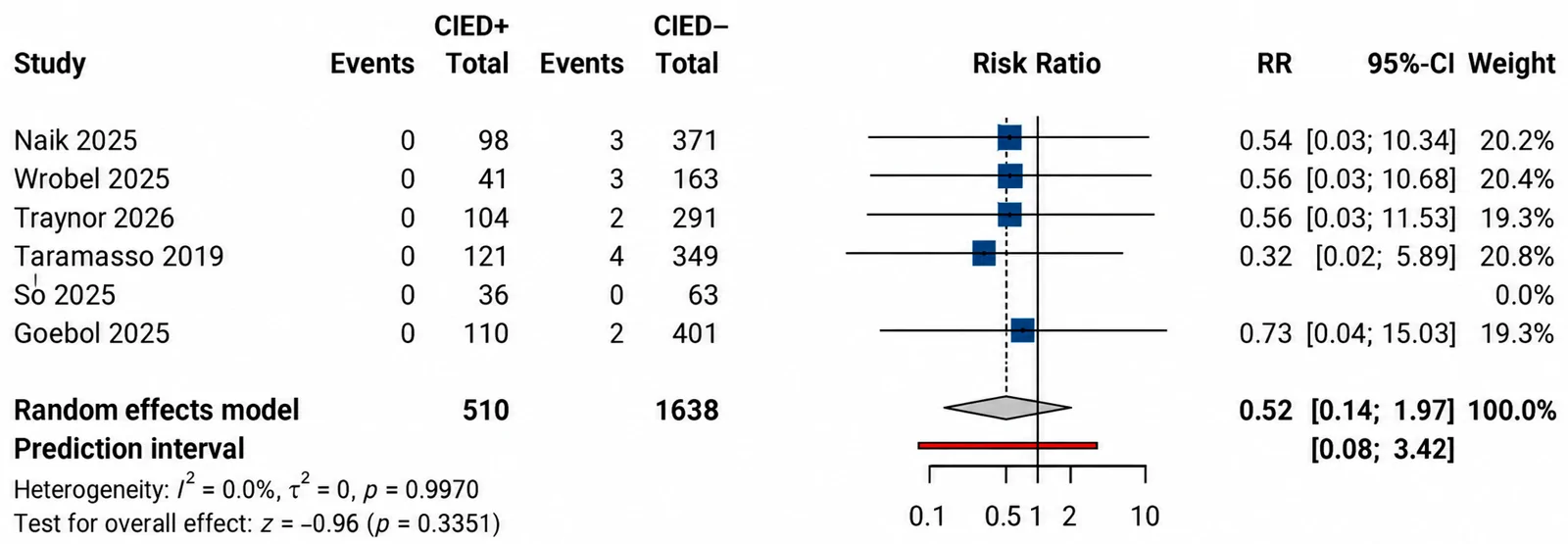
Forest plot of stroke risk comparing CIED versus no CIED.

**Figure 7:**
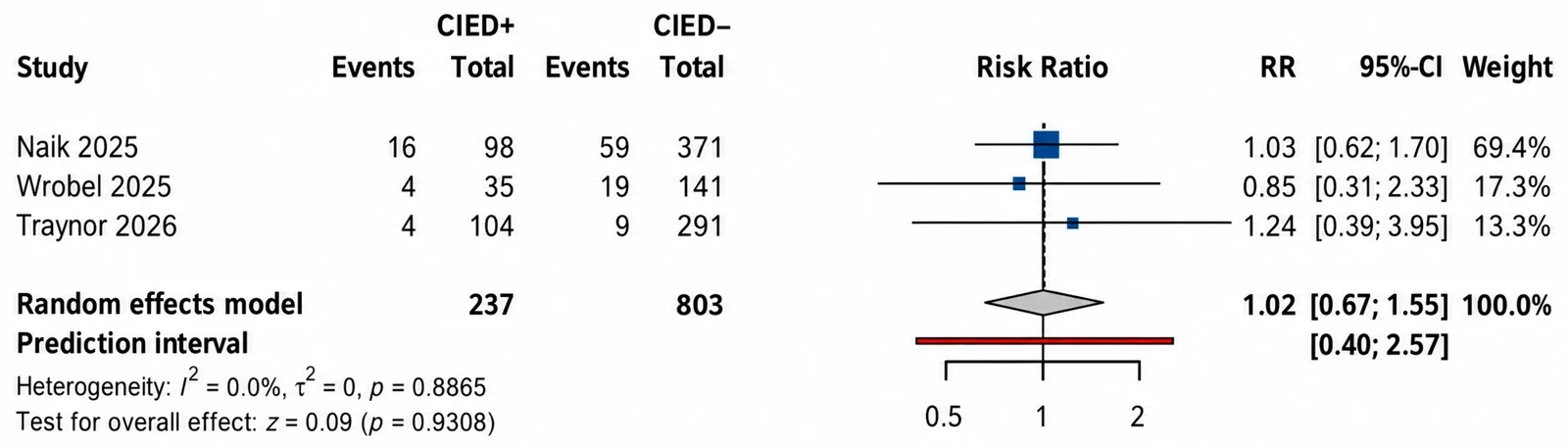
Forest plot of hospitalization for heart failure risk comparing CIED versus no CIED.

**Figure 8:**
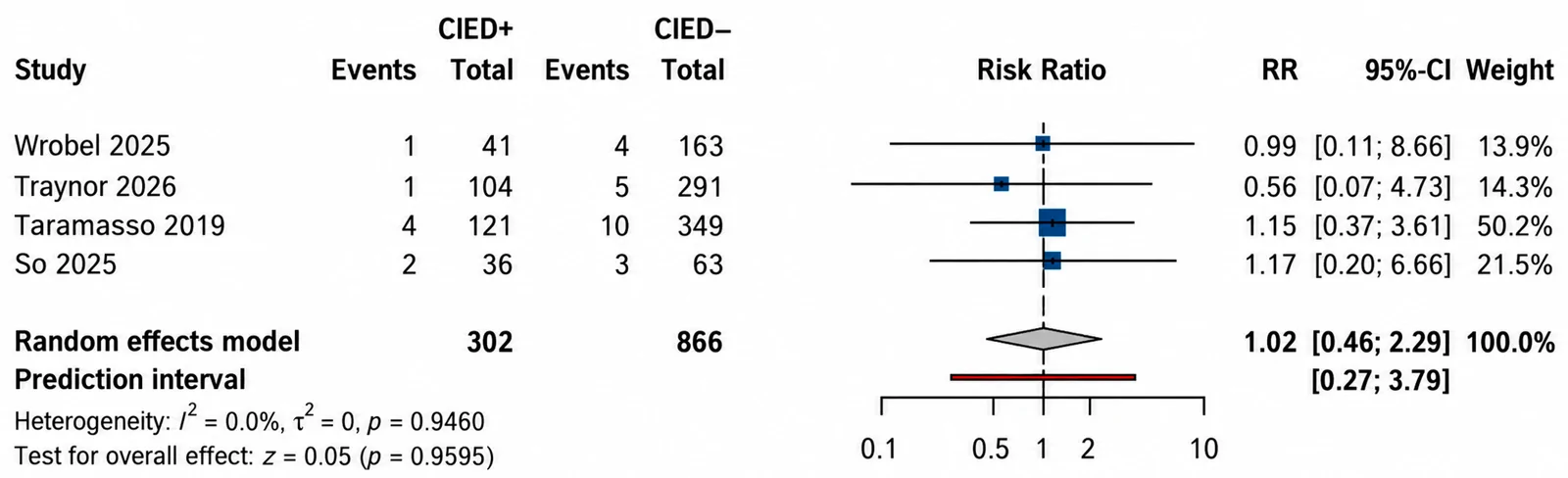
Forest plot of in-hospital mortality risk comparing CIED versus no CIED.

**Figure 9:**
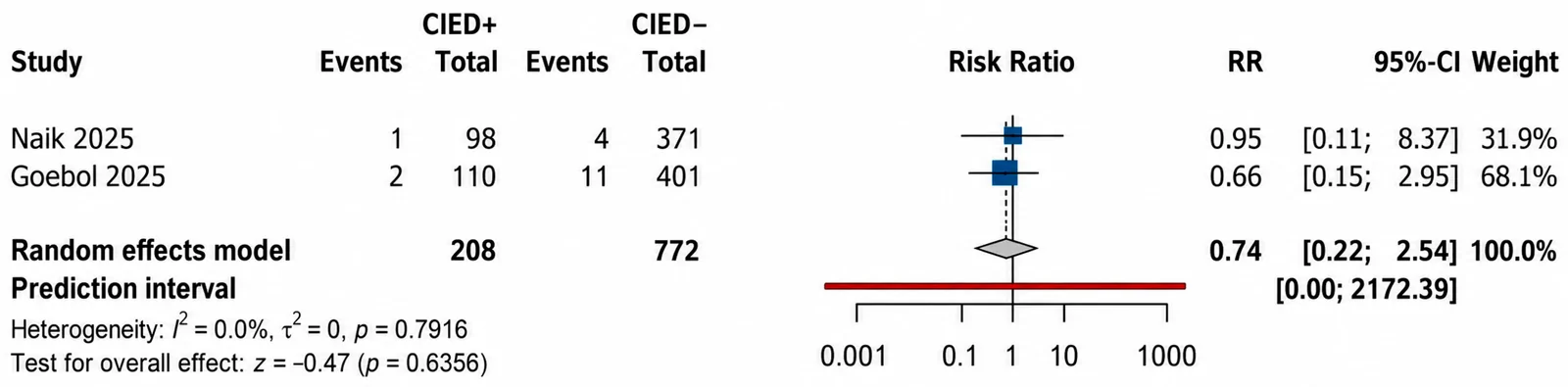
Forest plot of major adverse events risk comparing CIED versus no CIED.

**Figure 10:**
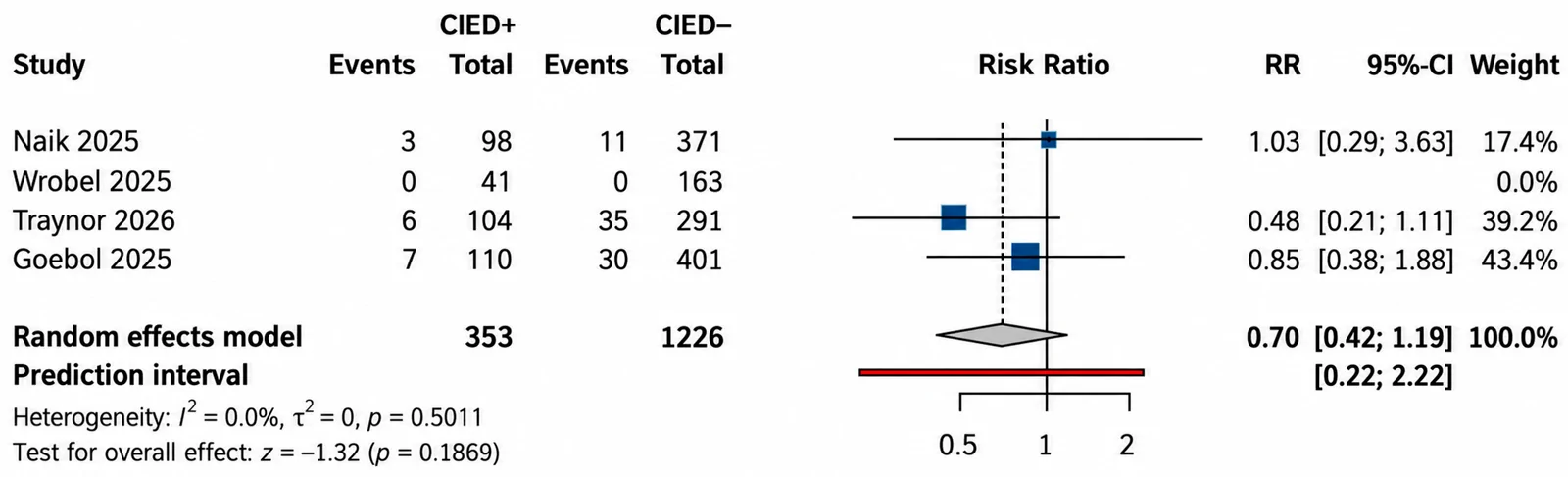
Forest plot of major bleeding risk comparing CIED versus no CIED.

**Figure 11:**
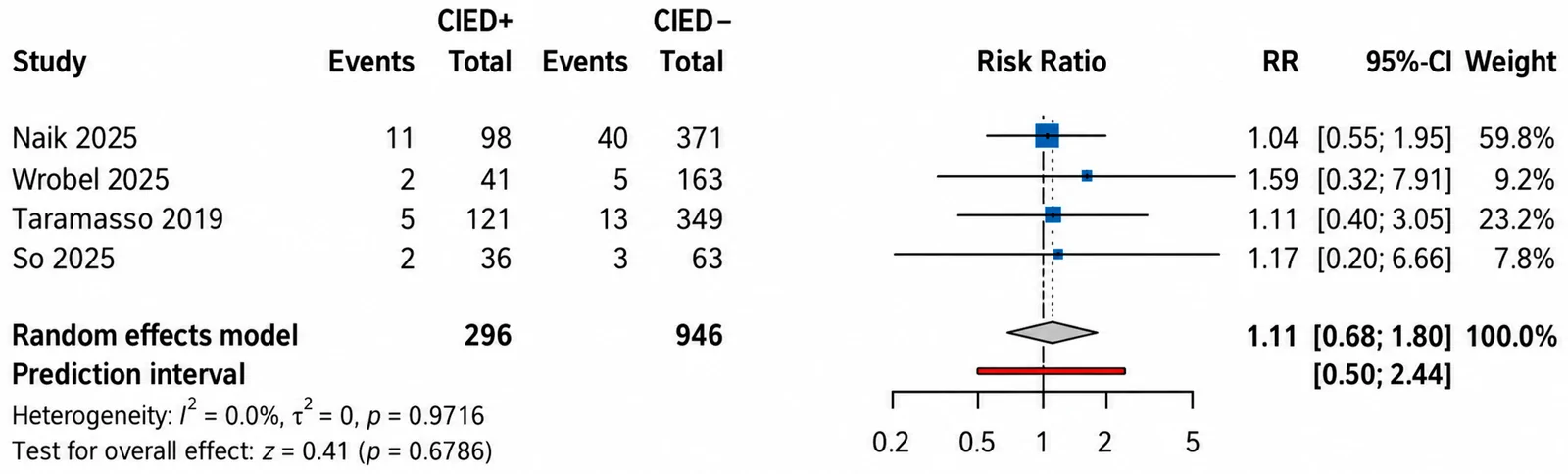
Forest plot of mortality risk comparing CIED versus no CIED.

**Figure 12:**
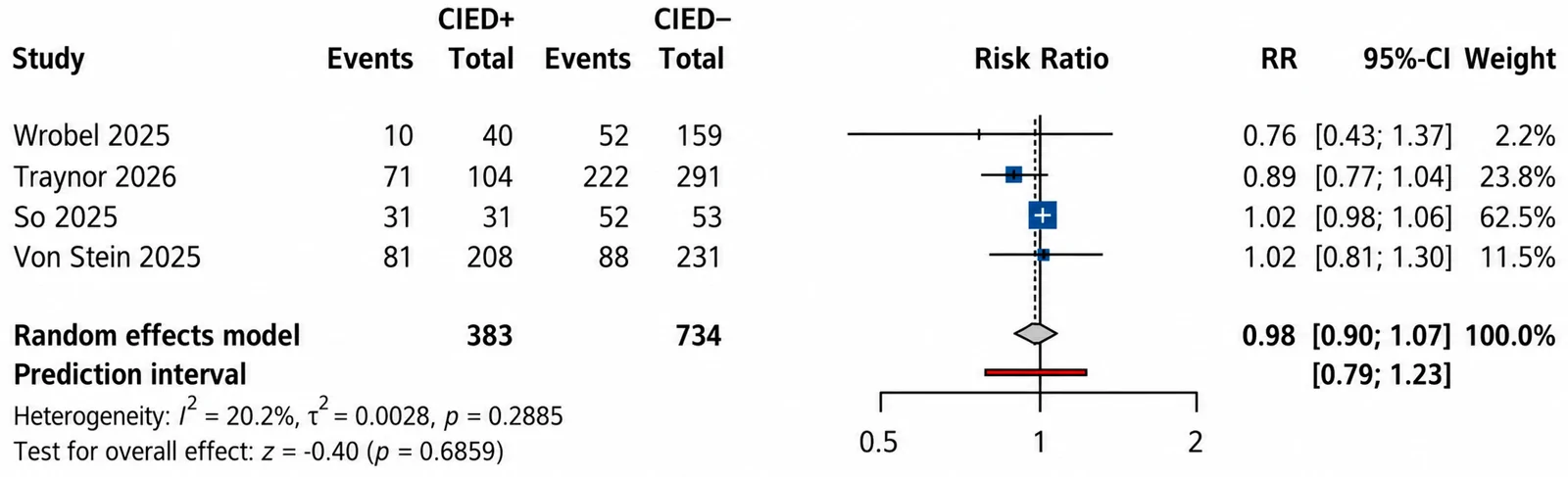
Forest plot of mild residual tricuspid regurgitation risk comparing CIED versus no CIED.

**Figure 13:**
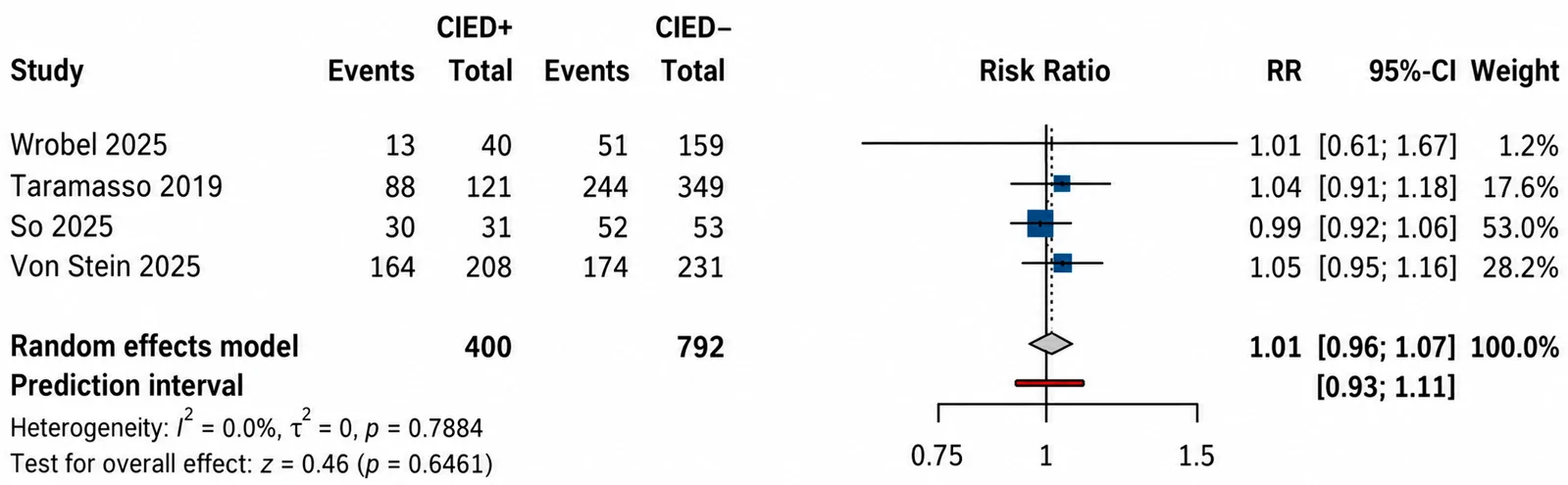
Forest plot of moderate residual tricuspid regurgitation risk comparing CIED versus no CIED.

**Table 1:** Baseline Characteristics of Included Studies.

Study Identifier	Protocol Registration Number	Country	Study Type	Arm	No.	Female		Age (yrs)		Mean Follow-Up (Months)	KCCQ Score		NYHA Functional Class III or IV		EuroSCORE II		TRI-SCORE		TR Severity No. (%)			AF No. (%)	HTN No. (%)	HF Hospitalization No. (%)	Diabetes No. (%)	CAD No. (%)	6MWD, m	
						No.	%	Mean	SD		Mean	SD	No.	%	Mean	SD	Mean	SD	Severe	Massive	Torrential						Mean	SD
TRILUMINATE	NCT03904147	USA, Canada, Germany, Italy	Observational subgroup analysis of an RCT	CIED+	98	58	59.2	80.2	8.6	24	51.9	21	62	63.3	NR	NR	NR	NR	NR	NR	NR	89 (90.8)	82 (83.7)	27 (27.6)		NR	222.8	121.5
				CIED−	371	223	60.1	78.2	7.6		55	23.1	208	56.1	NR	NR	NR	NR	NR	NR	NR	314 (84.6)	298 (80.3)	92 (24.8)	NR	NR	238.3	118.9
Wrobel 2025	NR	Germany	Retrospective Cohort	CIED+	41	11	26.83	79	6.14	1	NR	NR	38	92.68	6.99	6.34	25.56	18.91	11 (26.83)	12 (29.27)	18 (43.9)	39 (95.12)	NR	NR	11(26.83)	17(41.46)	NR	NR
				CIED−	163	128	78.53	78.33	5.98		NR	NR	145	88.95	5.34	5.57	19.89	15.68	60 (36.81)	41 (25.15)	62 (38.03)	144 (88.34)	NR	NR	41(25.15)	59(36.2)	NR	NR
Euro TR	NCT06307262	Germany	Retrospective Cohort	CIED+	851	378	44.6	79.66	5.198	60	NR	NR	727	85.4	5.4	4	5.6	2.22	338/821 (41.2)	277/821 (33.7)	190/821 (23.1)	757/839 (90.2)	641/807 (79.4)	273/450 (60.7)	209/806 (25.9)	393/843 (46.6)	237.26	139
				CIED−	2174	1267	58.3	79.66	5.192		NR	NR	1782	81.9	4.7	3.6	5.3	2.22	979/2100 (46.6)	674/2100 (32.1)	390/2100 (18.6)	1934/2145 (90.2)	1594/2030 (78.5)	604/1092 (55.3)	450/2006 (22.4)	832/2120 (39.2)	251	123.8
TRIPLACE	NCT06033274	USA, Canada, Denmark, France	Retrospective Cohort	CIED+	104	57	54.8	77.79	1.56	12	NR	NR	81	77.9	5.8	4.51	5.33	2.25	36 (34.6)	29 (27.9)	39 (37.5)	92 (88.5)	72 (69.2)	49 (47.6)	23 (22.1)	49 (47.1)	226.6	101.49
				CIED−	291	194	66.7	76.03	0.98		NR	NR	224	77.2	4.3	2.83	5	1.49	79 (27.1)	89 (30.6)	123 (42.3)	251 (86.3)	194 (66.7)	126 (43.3)	63 (21.6)	100 (34.4)	236.25	122.74
TriValve	NCT03416166	Switzerland	Prospective Cohort	CIED+	121	54	44	75	9	12	NR	NR	116	95.5	10.8	10	NR	NR	NR	NR	NR	50 (40.0)	NR	106 (87.8)	NR	NR	NR	NR
				CIED−	349	208	59	77.7	8.6		NR	NR	322	92.3	10.3	5	NR	NR	NR	NR	NR	120 (35.0)	NR	240 (69.0)	NR	NR	NR	NR
So 2025	NR	17 international centers	Retrospective Cohort	CIED+	36	17	47.2	77.4	8.4	1	NR	NR	36	100	7.13	5	6.4	2.6	NR	36	NR	33 (91.7)	29 (80.6)	NR	8 (22.2)	17 (47.2)	NR	NR
				CIED−	63	32	50.8	74.3	9		NR	NR	63	100	4.13	2.8	5.5	2.5	NR	63	NR	56 (88.9)	39 (61.9)	NR	13 (20.6)	17 (27)	NR	NR
bRIGHT EU PAS	NCT04483089	26 sites in Europe	Prospective Cohort	CIED+	110	48	43.6	78.7	7.8	1	42.32	24.37	90	82.6	NR	NR	NR	NR	13 (12)	66 (60.2)	30 (26.9)	95 (86.4)	96 (87.3)	54 (49.1)	28 (25.5)	NR	NR	NR
				CIED−	401	237	59.1	79	7		45.1	22.05	317	79.1	NR	NR	NR	NR	38 (9.4)	247 (61.6)	107 (26.7)	346 (86.3)	347 (86.3)	160 (39.9)	86 (21.4)	NR	NR	NR
Braunstein 2025	NR	USA	Retrospective Cohort	CIED+	21	11	52.4	80	7.8	12	NR	NR	NR	NR	NR	NR	NR	NR	NR	NR	NR	17 (85.0)	16 (76.2)	NR	4 (19.1)	12 (57.1)	NR	NR
				CIED−	42	31	73.8	75.3	10.1		NR	NR	NR	NR	NR	NR	NR	NR	NR	NR	NR	33 (80.5)	21 (50.0)	NR	13 (31.7)	18 (42.9)	NR	NR
Alachkar 2023	NR	Switzerland, Germany	Retrospective Cohort	CIED+	25	11	44	80.6	7.3	NR	NR	NR	17	NR	NR	NR	NR	NR	14 (56)	11 (44)	0	21 (84)	24 (96)	NR	10 (40)	18 (72)	NR	NR
				CIED−	81	53	65.4	79.9	6.1		NR	NR	54	NR	NR	NR	NR	NR	46 (56.8)	31 (38.3)	4 (4.9)	73 (90.1)	64 (79)	NR	19 (23.5)	42 (51.9)	NR	NR
Mekary 2026	NR	Georgia	Retrospective Cohort	TTVR cohort; single-arm study used for narrative synthesis only	73	48	66	71.5	15.3	10.5	NR	NR	NR	NR	NR	NR	NR	NR	NR	NR	NR	59 (81)	40 (55)	NR	19 (26)	16 (22)	NR	NR

RCT: Randomized Controlled Trial; No.: Number; yrs: years; SD: Standard Deviation; KCCQ: Kansas City Cardiomyopathy Questionnaire; NYHA: New York Heart Association; EuroSCORE II: European System for Cardiac Operative Risk Evaluation II; TRI-SCORE: Tricuspid Regurgitation Severity Score; TR: Tricuspid Regurgitation; AF: Atrial Fibrillation; HTN: Hypertension; HF: Heart Failure; CAD: Coronary Artery Disease; 6MWD: 6-Minute Walk Distance; m: meters; CIED: Cardiac Implantable Electronic Device; CIED+: Patients with a CIED; CIED−: Patients without a CIED; TTVR: Transcatheter Tricuspid Valve Replacement; NR: Not Reported.

## Data Availability

All data generated or analysed during this study are included in this published article and its Supplementary Information files.

## Supplementary Materials

The following supporting information can be downloaded at https://www.mdpi.com/article/10.3390/jcdd13100478/s1. Table S1: NIH quality assessment of observational cohort and cross-sectional studies.

## Abbreviations

ACS	Acute Coronary Syndrome
AF	Atrial Fibrillation
CAD	Coronary Artery Disease
CAVI	Caval Valve Implantation
CI	Confidence Interval
CIED	Cardiac Implantable Electronic Device
CRT	Cardiac Resynchronization Therapy
CRTD	Cardiac Resynchronization Therapy Defibrillator
CVD	Cardiovascular Death
ICD	Implantable Cardioverter Defibrillator
KCCQ	Kansas City Cardiomyopathy Questionnaire
MAE	Major Adverse Event
MeSH	Medical Subject Headings
NIH	National Institutes of Health
NYHA	New York Heart Association
PRISMA	Preferred Reporting Items for Systematic Reviews and Meta-Analyses
PROSPERO	International Prospective Register of Systematic Reviews
RCT	Randomized Controlled Trial
REML	Restricted Maximum Likelihood
RR	Risk Ratio
SLDA	Single-Leaflet Device Attachment
TEER	Transcatheter Edge-to-Edge Repair
TLE	Transvenous Lead Extraction
TR	Tricuspid Regurgitation
TRI-SCORE	Tricuspid Regurgitation International Risk Score
TTVA	Transcatheter Tricuspid Valve Annuloplasty
TTVI	Transcatheter Tricuspid Valve Intervention
TTVR	Transcatheter Tricuspid Valve Replacement
6MWD	Six-Minute Walk Distance
