# Supplementary material for: The Impact of Preexisting Cardiac Implantable Electronic Devices on the Safety of Transcatheter Tricuspid Valve Interventions: A Systematic Review and Meta-Analysis
Source: J Cardiovasc Dev Dis. 2026 Sep 23;13(10):478. doi: 10.3390/jcdd13100478 (PMC13705483; doi:10.3390/jcdd13100478)
Supplement: Supplementary file 1 [file jcdd-13-00478-s001.zip › jcdd-4436444-supplementary.pdf]

Table S1. NIH quality assessment of observational cohort and cross-sectional studies

ID	NIH Quality Assessment Tool for Observational Cohort and Cross-Sectional Studies														total scores	Quality rating: good (11-14 points) or fair (7.5-10.5 points) or poor (0-7 points)
	1. Was the research question or objective in this paper clearly stated?	2. Was the study population clearly specified and defined?	3. Was the participation rate of eligible persons at least 50%?	4. Were all the subjects selected or recruited from the same or similar populations (including the same time period)? Were inclusion and exclusion criteria for being in the study	5. Was a sample size justification, power description, or variance and effect estimates provided?	6. For the analyses in this paper, were the exposure(s) of interest measured prior to the outcome(s) being measured?	7. Was the time frame sufficient so that one could reasonably expect to see an association between exposure and outcome if it existed?	8. For exposures that can vary in amount or level, did the study examine different levels of the exposure as related to the outcome (eg, categories	9. Were the exposure measures (independent variables) clearly defined, valid, reliable, and implemented consistently across all study participants?	10. Was the exposure(s) assessed more than once over time?	11. Were the outcome measures (dependent variables) clearly defi ned, valid, reliable, and implemented consistently across all study participants?	12. Were the outcome assessors blinded to the exposure status of participants?	13. Was loss to follow-up after baseline 20% or less?	14. Were key potential confounding variables measured and adjusted statistically for their impact on the relationship between exposure(s) and outcome(s)?		
	Yes / No / Not reported (NR) or cannot determine (CD) or not applicable (NA)	Yes / No / Not reported (NR) or cannot determine (CD) or not applicable (NA)	Yes / No / Not reported (NR) or cannot determine (CD) or not applicable (NA)	Yes / No / Not reported (NR) or cannot determine (CD) or not applicable (NA)	Yes / No / Not reported (NR) or cannot determine (CD) or not applicable (NA)	Yes / No / Not reported (NR) or cannot determine (CD) or not applicable (NA)	Yes / No / Not reported (NR) or cannot determine (CD) or not applicable (NA)	Yes / No / Not reported (NR) or cannot determine (CD) or not applicable (NA)	Yes / No / Not reported (NR) or cannot determine (CD) or not applicable (NA)	Yes / No / Not reported (NR) or cannot determine (CD) or not applicable (NA)	Yes / No / Not reported (NR) or cannot determine (CD) or not applicable (NA)	Yes / No / Not reported (NR) or cannot determine (CD) or not applicable (NA)	Yes / No / Not reported (NR) or cannot determine (CD) or not applicable (NA)	Yes / No / Not reported (NR) or cannot determine (CD) or not applicable (NA)		
Naik 2025 (TRILUMINATE CIED subgroup)	Yes	Yes	NR	Yes	No	Yes	Yes	NA	Yes	No	Yes	No	NR	No	9	Fair
Wrobel 2025	Yes	Yes	Yes	Yes	No	Yes	No	NA	Yes	CD	Yes	NR	No	NR	8.5	Fair
von Stein 2025	Yes	Yes	Yes	Yes	No	Yes	Yes	NA	Yes	No	Yes	No	NR	NR	9.5	Fair
Traynor 2026	Yes	Yes	No	Yes	No	Yes	Yes	NA	Yes	No	Yes	No	Yes	No	10.5	Fair
Taramasso 2020 (TriValve)	Yes	Yes	Yes	Yes	No	Yes	Yes	NA	Yes	No	Yes	No	Yes	No	11	Good
So 2025	Yes	Yes	Yes	Yes	No	Yes	No	NA	Yes	No	Yes	No	Yes	No	10.5	Fair
Goebel 2025	Yes	Yes	Yes	Yes	No	Yes	Yes	NA	Yes	No	Yes	No	Yes	No	11	Good
Braunstein 2025	Yes	Yes	Yes	Yes	No	Yes	Yes	NA	Yes	No	CD	No	Yes	No	10	Fair
Alachkar 2023	Yes	Yes	Yes	Yes	No	Yes	No	NA	CD	No	CD	No	CD	No	7.5	Fair
Mekary 2026	Yes	Yes	CD	Yes	No	Yes	CD	NA	CD	No	CD	No	CD	No	6	Poor

Note: Naik et al. was assessed with the NIH tool because the CIED-positive versus CIED-negative comparison was not randomized. NR, not reported; CD, cannot determine; NA, not applicable.
